# Incidence and risk factors for new-onset diabetes mellitus (NODM) in post-pancreatectomy patients diagnosed with pancreatic adenocarcinoma (PAC): A systematic review

**DOI:** 10.18632/oncoscience.637

**Published:** 2025-12-08

**Authors:** Adavikolanu Kesava Ramgopal, Chandramouli Ramalingam, Kaliyath Soorej Balan, K.S. Abhishek Raghava, Kondapuram Manish, Kari NagaSai Divya, Yadala Ambedkar, Varthya Shobhan Babu, Kondeti Ajay Kumar

**Affiliations:** ^1^Department of Radiation Oncology, All India Institute of Medical Sciences (AIIMS), Mangalagiri, India; ^2^Department of Radiation Oncology, AIIMS, Bibinagar, Hyderabad, India; ^3^Department of Medical Oncology, AIIMS, Mangalagiri, India; ^4^Department of Radiation Oncology, JIPMER, Pondicherry, India; ^5^Department of Pharmacology, AIIMS, Jodhpur, India

**Keywords:** pancreatectomy, pancreatic adenocarcinoma, diabetes mellitus

## Abstract

Background: Pancreatic adenocarcinoma is a highly aggressive malignancy often requiring pancreatectomy as part of curative treatment. However, pancreatectomy frequently leads to endocrine dysfunction, such as new-onset diabetes mellitus (NODM). But the relationship between post pancreatectomy NODM and pancreatic carcinoma and the relevant risk factors remains underexplored.

Methods: In accordance with the PRISMA guidelines, a systematic search for pertinent studies was conducted across electronic databases including MEDLINE, Cochrane, EMBASE, and Scopus, covering the period from January 2000 to March 2025. The quality of these studies was evaluated using the Newcastle-Ottawa Scale, specifically designed for cohort studies. Subgroup analysis was done in terms of different pancreatectomy procedures.

Results: 45 quantitative studies were analysed, of which 16 (35.5%) were prospective studies and 29 (64.5%) were retrospective studies. Regarding the subgroup analysis, 11 studies analysed Pancreatico-Duodenectomy (PD) alone, another 12 studies analysed Distal Pancreatectomy (DP) alone, and the rest of the 22 studies compared PD with DP. The overall incidence of NODM was 24.5%, with the PD group incidence being 23.2%, and the DP group incidence was 26.3%. Older age, High BMI, preop hyperglycemia, pre-op high HbA1c, pre-existing chronic pancreatitis, low remnant pancreatic volume and post-operative complications were associated with a high incidence of NODM.

Conclusions: The development of NODM after partial pancreatic resections for pancreatic adenocarcinoma is a severe complication that requires prompt diagnosis, careful monitoring and systematic management. Hence, healthcare professionals should have detailed knowledge of the surgical procedure and its potential for diabetes complications postoperatively, using risk factor assessment.

## INTRODUCTION

Pancreatic cancer is among the deadliest types of cancer, characterized by its insidious onset, aggressive behavior, with poor outcomes. According to estimates from GLOBOCAN 2020, pancreatic cancer continues to contribute significantly to the global disease burden, ranking as the 12th most prevalent cancer (2.6% of all cancers) and the 7th leading cause of cancer-related deaths (4.7% of all cancers) [1]. Pancreatic adenocarcinoma constitutes 90% of all known pancreatic cancer cases [2].

The management of Pancreatic Adenocarcinoma include various options such as surgery, chemotherapy, radiotherapy and newer molecular therapies such as targeted therapy, immunotherapy etc. Nevertheless, the primary treatment approach continues to be surgical resection and include procedures such as Pancreaticoduodenectomy (PD) also known as the Whipple procedure, distal pancreatectomy (DP) which involves the tail or tail and a portion of the body, central pancreatectomy (CP), and total pancreatectomy (TP) [3, 4].

With rapid progress in imaging technology, it has enabled earlier identification and removal of pancreatic tumors [5]. As a result of diagnosing lesions at a stage suitable for surgery sooner, the frequency of pancreatic surgical procedures has also risen [5, 6]. The survival rates following pancreatectomy for cancer have improved, probably due to earlier cancer detection, advancements in surgical methods, the concentration of pancreatic surgeries in high-volume hospitals, and with ready adoption of new chemotherapy drugs [7, 8].

A retrospective analysis of 959 patients who had pancreatic adenocarcinoma resection indicated survival rates of 19% at 5 years and 10% at 10 years following the surgery [9]. Consequently, the decline in both endocrine and exocrine functions can significantly affect the quality of life for the growing population of patients following pancreatectomy. Therefore, it is essential to prioritize the preservation of endocrine and exocrine functions during pancreatectomy [10].

New-onset diabetes mellitus (NODM) in patients without diabetes following surgery was classified based on the diagnostic criteria set by the American Diabetes Association (ADA), namely: (a) an HbA1c level of ≥6.5 %, (b) a fasting plasma glucose level of ≥126 mg/dl after at least 8 hours of fasting, or (c) a 2-hour plasma glucose level of ≥200 mg/dl during an oral glucose tolerance test [11]. NODM was defined as diagnosis of DM within two years of diagnosis of pancreatic carcinoma [12]. Pancreatic resection is a recognized cause of NODM, due to loss of islet mass [13].

Regarding post-pancreatectomy diabetes, the extent of endocrine insufficiency may be associated with the specific area of the pancreas that was removed, with distal pancreatectomy thought to have a greater likelihood of leading to glucagon deficiency and hypoglycemia because of the differences in islet cell distribution, predominantly located in the pancreatic body and tail [14, 15].

Regarding pancreaticoduodenectomy procedure, surgical removal of the pylorus and/or duodenum is expected to influence the action of incretins and the regulation of blood sugar. Studies indicate that these individuals exhibit heightened secretion of GLP-1, lowered levels of GIP, and decreased insulin secretion [16].

Multiple complications have been reported following the resection of different quantities of pancreatic tissue. Among these complications, endocrine insufficiency, including new-onset diabetes mellitus (NODM), is particularly challenging as it is closely linked to the prognosis of pancreatic cancer [17].

Since NODM can place a significant strain on patients, their families, and healthcare resources, it is crucial to determine how often NODM occurs and its severity, as well as to understand how diabetes impacts patients’ quality of life and overall lifespan.

Hence, to address these issues, we are conducting a systematic review to determine the incidence as well as risk factors associated with NODM in pancreatic adenocarcinoma, post resection.

## RESULTS

### Literature search

Out of the 1479 articles searched, 972 articles remained in the dataset after eliminating duplicates. We excluded 643 records that focused on individual case reports, reviews, specific tumor types, surgical methods, various postoperative complications, and studies on non-malignant and benign lesions. The full texts of 329 articles were reviewed, and 247 of these were discarded due to reasons such as being conference abstracts, duplicate studies, non-English publications, study protocols, research on total pancreatectomy, lacking information on long-term postoperative complications, or detailing surgical methods.

**Figure 1:**
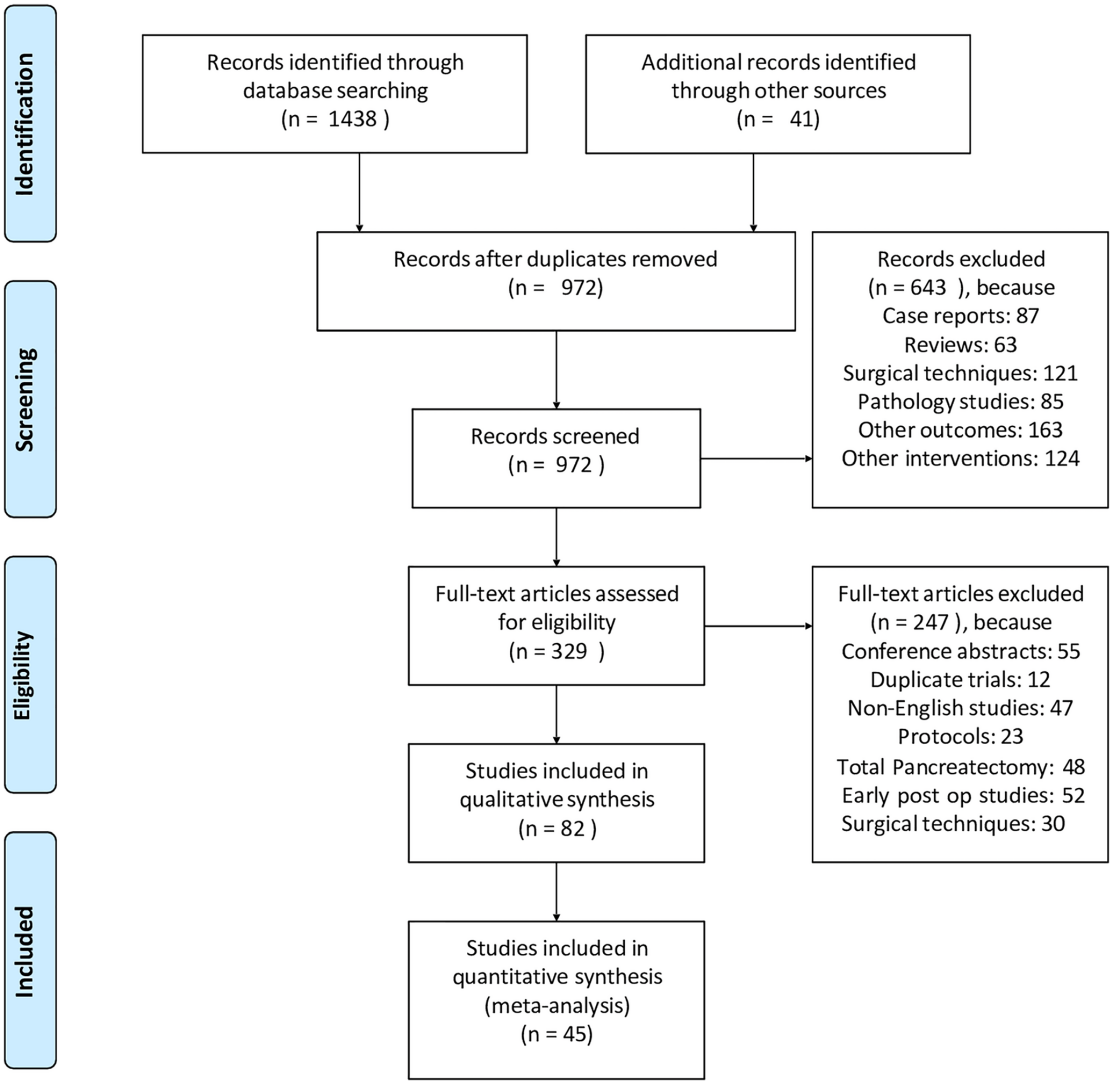
PRISMA flow diagram of the study collection. Source Moher et al.

82 remaining articles were eligible, out of which 15 were eliminated because they either failed to report the patients’ preoperative diabetes status or had an inadequate number of participants. Additionally, 22 studies were omitted from the final evaluation due to poor quality or insufficient patient numbers regarding the study outcome. In the end, 45 studies involving pancreatic cancer patients who underwent either PD or DP were included in the quantitative analysis (Figure 1).

### Study characteristics and quality assessment

45 quantitative studies were chosen for this review (Table 1). Out of these 45 cohort studies, 11 studies included Pancreaticoduodenectomy (PD) alone, with 6 of them being prospective studies, while the remaining 5 studies were retrospective. 12 studies included Distal Pancreatectomy (DP) alone, with 4 of them being prospective studies, while the remaining 8 studies were retrospective. The remaining 22 studies included both PD and DP procedures, with 6 of them being prospective studies, while the remaining 16 studies were retrospective.

**Table 1:** NODM post pancreatectomy: Summary of the studies included in the systematic review and meta-analysis

No.	References	Year	Country	Total patients (*n*)	% PAC	Age	Male; Female	Study quality and Score	NODM (%)
**Pancreaticoduodenectomy (PD)-Prospective studies**									
1	Kanwat et al. [18]	2023	India	64	88	Median 54	59;41	High (6)	22
2	Niwano et al. [19]	2022	Japan	96	67.7	66.3 ± 1.12	53;47	High (6)	34.3
3	Maxwell et al. [20]	2019	USA	403	65	59.9 ± 13.6	21;79	High (6)	16.6
4	Singh et al. [21]	2018	India	50	15	50.0 ± 10.7 (no DM), 53.3 ± 8.5 (DM)	30;70	High (5)	22
5	Yun et al. [22]	2017	South Korea	66	90.9	62.0 ± 11.3 years	43;57	High (6)	24.2
6	Pannala et al. [23]	2008	USA	512	100	66 ± 11 years	72;28	Moderate (4)	74
**Pancreaticoduodenectomy (PD)-Retrospective studies**									
7	Wu et al. [24]	2015	Taiwan	4775	44	3 groups: ≤49, 50–64, ≥65	55;45	High (6)	15.1
8	Min Oh et al. [25]	2013	South Korea	98	92.9	Mean: 66.2 ± 11.8	61;39	Moderate (4)	17.4
9	Ferrera et al. [26]	2012	Italy	564	75	Mean 64	55:45	High (5)	4
10	Bock et al. [27]	2012	Germany	90	53.3	Median 64 (range 28–91)	51;49	Moderate (4)	19
11	You et al. [28]	2011	South Korea	55	71	Median: 60 (range 14–76)	64;26	Moderate (4)	35
**Distal Pancreatectomy (DP)- Prospective studies**									
12	Imamura et al. [29]	2023	Japan	56	55.4	65.9 ± 1.5 years	34;66	High (6)	54.8
13	Tariq et al. [30]	2019	India	216	21.75	No-DM: 56 ± 15.9, Pre-DM: 65 ± 13.8	46;54	High (6)	51.06
14	Kang et al. [31]	2015	South Korea	101	20.8	Mean 54.1 ± 12.7	37;63	High (6)	47.6
15	Shirakawa et al. [32]	2012	Japan	61	41	Mean 62 ± 14	39;61	High (5)	36
**Distal Pancreatectomy (DP)- Retrospective studies**									
16	Shen et al. [33]	2023	China	194	44	58 ± 12.9	43;57	Moderate (4)	24.7
17	Izumo et al. [34]	2020	Japan	150	11	Median 60 (range 19–83)	36;64	High (6)	39
18	Wu et al. [35]	2018	Taiwan	1980	13.2	Mean: 51.5 ± 16.3 years	48;52	High (6)	24.7
19	Kwon et al. [36]	2016	South Korea	111	20.7	Mean: 50.0 ± 1.4 years	30;70	High (6)	14.1
20	Malleo et al. [37]	2014	Italy	100	4	Mean: 47.0 ± 15.4	53;47	High (5)	13.0
21	Stuthfield et al. [38]	2009	UK	65	92	Mean: 49.9 (range 15–88)	41;59	High (5)	17
22	Chari et al. [39]	2008	USA	736	100	Mean 68.5 ± 11.2 (cases)	50;50	High (6)	52.3
23	Adam et al. [40]	2001	USA	85	65	54 ± 13.7	49;51	High (5)	18
**PD & DP- Prospective studies**									
24	Lee et al. [41]	2023	South Korea	224 (PD: 152, DP: 72)	66.5	PD: 64.8 ± 10.4, DP: 59.7 ± 13.5	53;47	High (6)	PD: 13, DP: 36
25	Ishida et al. [42]	2021	Japan	69 (PD: 40, DP: 29)	30	Median 65 (PD), 63 (DP)	47;53	High (5)	PD: 17.5, DP: 27.6
26	Shaw et al. [43]	2021	UK	80	39	Mean 64 (range 37.8–77.9)	46;54	High (6)	PD:20.5, DP: 28.75
27	Niwano et al. [44]	2021	Japan	109 (PD: 73, DP: 36)	66.1	Mean 66.1 ± 0.87	57;43	High (6)	PD: 1.4, DP:8.3
28	Maxwell et al. [45]	2020	USA	1083	19.9	Mean 62 ± 13.2	53;47	High (6)	PD: 27.2, DP: 39.9
29	Maignan et al. [46]	2018	France	91 (PD: 65, LP: 26)	46.2	Median 66.3 (range 17–86)	58;42	High (5)	PD:9.1, DP:10
**PD & DP- Retrospective studies**									
30	Wang et al. [47]	2025	China	509	100	Mean: 63.2 ± 11.0	51.8;48.2	High (6)	PD: 52.8, DP: 83.6
31	Yoo et al. [48]	2023	South Korea	30,242	100	Mean: 61.5 ± 9.0	53;47	High (6)	PD: 7, DP: 14.2
32	Schranz et al. [49]	2023	Austria	370	84.3	Mean: 69 (malignant 70, benign 63)	49.5;50.5	High (5)	PD: 12.2, DP: 9.5
33	Thomas et al. [50]	2022	Australia	4706	61.7	Grouped: <45, 45–65, ≥65	47.2;52.8	High (6)	PD: 13.8 DP: 24.7
34	Hamad et al. [51]	2022	USA	4255	79.9	Median 74 (IQR 69–79)	51.9;48.1	High (6)	PD: 61.1, DP: 38.9
35	Yamada et al. [52]	2021	Japan	403	70.20	66 ± 9.4	44.7;55.3	Moderate (4)	PD: 21, DP: 25
36	Yamamoto et al. [53]	2020	Japan	681	54.9	Median: 70 (IQR 63–77)	58.9;41.1	High (6)	PD: 18.4, DP: 21.1
37	Mayeux et al. [54]	2020	France	80	40	Median: 64.3 yrs (IQR: 37.9–77.9)	37;63	Moderate (4)	PD: 25, DP: 29.4
38	Lee et al. [55]	2020	Australia	137	64.2	Mean: 64.2 ± 12.0	51;49	High (5)	PD: 22.2, DP: 28.2
39	Kusakabe [56]	2019	Japan	1717	74.4	Mean: 62.6 ± 12.8	51.8;48.2	High (6)	PD: 20.4, DP: 10.1
40	Karlin et al. [57]	2018	USA	184 (92 DM + 92 non-DM)	88	Mean: 69.5 ± 9.0 years	52;48	High (6)	PD: 24, DP: 26
41	Nguyen et al. [58]	2017	USA	472 (DP:122, PD:350)	63.1	Mean: 61.5 ± 7.0 years	53;47	High (6)	PD: 43, DP: 45
42	Elliott et al. [59]	2017	USA	1165	59.7	Median 62.5 (IQR 52.5–70.9)	541;34	High (6)	PD: 40.4, DP: 36.2
43	Burkhart et al. [60]	2015	USA	259	30	PD: Median 66, DP: Median 64	38;62	High (6)	PD: 18, DP: 31
44	Hirata et al. [61]	2014	Japan	167 (PD: 100, DP: 67)	40.1	Mean 66.0 ± 12.2 years	67;33	High (5)	PD: 24.2, DP: 31.2
45	White et al. [62]	2011	Australia	101	100	Mean: 64.3 ± 10.7	55;45	Moderate (4)	PD: 9.6, DP: 9.5

Out of the 45 cohort studies, the majority (*n* = 37) of studies had high quality, with eight (*n* = 08) studies having moderate quality critical appraisal scores, added in the Supplementary File 1 (Table).

### Incidence of NODM

Among the 45 included studies, the overall incidence of NODM in the studied population was 24.5%. Incidence rates calculated using the random-effects model, concurred with our study findings, the forest plots of which were included in the Supplementary File 2.

We further divided the studies into 2 subgroups according to the type of resection (PD or DP) done for pancreatic cancer cases. For patients who underwent PD, the incidence of NODM was 23.2%, and for DP resection cases, the NODM incidence was 26.3%.

### Analysis of risk factors for incidence of NODM

Around 37 risk factors were analysed in the 45 included studies, out of which 24 risk factors were associated with the incidence of NODM (Tables 2 and 3).

**Table 2:** NODM post pancreatectomy: Summary of the risk factors in the systematic review and meta-analysis

No.	References	Risk factors analysed	Risk factors associated with NODM
**PD-Prospective**			
1	Kanwat et al. [18]	Age, BMI, Chronic pancreatitis, Remnant pancreatic volume	Remnant pancreatic volume
2	Niwano et al. [19]	Age, Hyperglycemia, HbA1c, Insulin levels, C-peptide	Insulin levels
3	Maxwell et al. [20]	Age, BMI, Hyperglycemia, HbA1c, Insulin levels, C-peptide	Hyperglycemia, HbA1c
4	Singh et al. [21]	Hyperglycemia, HbA1c, Insulin levels, C-peptide, Remnant pancreatic volume	Remnant pancreatic volume
5	Yun et al. [22]	Tumor stage, Remnant pancreatic volume	Tumor stage, Remnant pancreatic volume
6	Pannala et al. [23]	Age, BMI, Family history	Age, BMI, Family history
**PD-Retrospective**			
7	Wu et al. [24]	Chronic pancreatitis, Co-morbidity	Chronic pancreatitis
8	Min Oh et al. [25]	Chronic pancreatitis	Chronic pancreatitis
9	Ferrera et al. [26]	Sex, Hyperglycemia, BMI, Tumor size, Operative time, Renal impairment, Remnant pancreatic volume	Hyperglycemia, Remnant pancreatic volume
10	Bock et al. [27]	Age, BMI, Adjuvant Chemotherapy, Adjuvant Radiotherapy	Age
11	You et al. [28]	Hyperglycemia, Chronic pancreatitis, Chemotherapy, Radiotherapy, Remnant pancreatic volume, Post-op complications	Hyperglycemia, Chemotherapy, Radiotherapy, Remnant pancreatic volume, Post-op complications
**DP-Prospective**			
12	Imamura et al. [29]	Age, Sex, BMI, Hyperglycemia, Insulin levels, Insulin resistance, Remnant pancreatic volume	Remnant pancreatic volume
13	Tariq et al. [30]	Hyperglycemia, HbA1c, Insulin levels, Insulin resistance, C-peptide, Chronic pancreatitis	Hyperglycemia, HbA1c, Chronic pancreatitis
14	Kang et al. [31]	Age, Sex, BMI, HbA1c, Insulin levels, C-peptide, Adjuvant Radiotherapy, Remnant pancreatic volume	Female gender, BMI, Remnant pancreatic volume
15	Shirakawa et al. [32]	Age, Sex, BMI, Hyperglycemia, HbA1c, Liver impairment, Dyslipidemia, Amylase, Remnant pancreatic volume, Blood loss, Adjuvant Chemotherapy, Post-op complications	HbA1c, Remnant pancreatic volume
**DP-Retrospective**			
16	Shen et al. [33]	Age, Sex, BMI, Hyperglycemia, Smoking status, Alcohol, Operative blood loss, Remnant pancreatic volume	Age, BMI, Hyperglycemia Remnant pancreatic volume
17	Izumo et al. [34]	Hyperglycemia, HbA1c, Insulin levels, Insulin resistance, Remnant pancreatic volume, Adjuvant Radiotherapy	Hyperglycemia, HbA1c, Insulin levels, Insulin resistance
18	Wu et al. [35]	Age, Sex, Income, Co-morbidity, Dyslipidemia, Chronic pancreatitis	Dyslipidemia, Chronic pancreatitis
19	Kwon et al. [36]	BMI, Tumor size, Tumor location, Chronic Pancreatitis	Tumor size
20	Malleo et al. [37]	Age, BMI, Tumor size, Lymph node status, Operative time, Post-op complications, Remnant pancreatic volume	Tumor size, Operative time
21	Stuthfield et al. [38]	Age, Sex, Post-op complications	Post-op complications
22	Chari et al. [39]	Age, Sex, BMI, Hyperglycemia, Smoking, Family History, Weight loss	Weight loss
23	Adam et al. [40]	Post-op complications	Post-op complications
**PDDP-Prospective**			
24	Lee et al. [41]	HbA1c, Dyslipidemia, Remnant pancreatic volume, Insulin levels, Adjuvant chemotherapy,	HbA1c, Adjuvant chemotherapy
25	Ishida et al. [42]	Hyperglycemia, HbA1c, Insulin resistance, C-peptide	Hyperglycemia, HbA1c, Insulin resistance, C-peptide
26	Shaw et al. [43]	Age, Sex, Race, Education, Employment, Marital status, Chemotherapy	Chemotherapy
27	Niwano et al. [44]	Sex, BMI, Insulin resistance, C-peptide, Resection type	Insulin resistance, Resection type
28	Maxwell et al. [45]	Age, Race, BMI, Hyperglycemia, HbA1c, Smoking, Resection type, Remnant pancreatic volume	Hyperglycemia, HbA1c
29	Maignan et al. [46]	Age, Sex, MPD size, Chemoradiation, Remnant pancreatic volume	Remnant pancreatic volume
**PDDP-Retrospective**			
30	Wang et al. [47]	Age, Hyperglycemia, BMI, Tumor size, Tumor staging, Smoking, Jaundice, Renal impairment, Dyslipidemia	Hyperglycemia, BMI, Tumor size, Tumor staging, Jaundice, Renal impairment, Dyslipidemia
31	Yoo et al. [48]	Sex, Hyperglycemia	Sex, Hyperglycemia
32	Schranz et al. [49]	Sex, BMI, Tumor size, Post-op complications	Post-op complications
33	Thomas et al. [50]	Age, BMI, Co-morbidity	Age, BMI, Co-morbidity
34	Hamad et al. [51]	Sex, BMI, Hyperglycemia, Family history, Race	Sex, Hyperglycemia, Family history
35	Yamada et al. [52]	Age, Sex, BMI, Hyperglycemia, HbA1c, Insulin levels, Insulin resistance, Dyslipidemia, Pre op Chemoradiotherapy, Remnant pancreatic volume	Remnant pancreatic volume
36	Yamamoto et al. [53]	Age, Sex, BMI, Hyperglycemia, HbA1c, Chronic pancreatitis, Dyslipidemia	Age, BMI, Hyperglycemia, HbA1c,
37	Mayeux et al. [54]	Age, Sex, Race, Post-op complications	Post-op complications
38	Lee et al. [55]	Age, BMI, Hyperglycemia, HbA1c, Family history, Smoking, Tumor size, Tumor staging, Renal impairment	Age, Family history, Renal impairment
39	Kusakabe [56]	Sex, BMI, Hyperglycemia, Race, Smoking, Family history, Alcoholism	Sex, BMI, Hyperglycemia, Smoking, Family history,
40	Karlin et al. [57]	BMI, Hyperglycemia, HbA1c, CA 19.9	BMI
41	Nguyen et al. [58]	Age, Race	Age, Race
42	Elliott et al. [59]	Age, Sex, BMI, Race, Chronic Pancreatitis, Co-morbidity	Chronic Pancreatitis, Co-morbidity
43	Burkhart et al. [60]	Hyperglycemia, HbA1c, Insulin	Hyperglycemia, HbA1c, Insulin
44	Hirata et al. [61]	Age, BMI, Jaundice, Albumin, Chronic Pancreatitis, Post-op complications	BMI, Chronic pancreatitis
45	White et al. [62]	BMI, Post-op complications	Post-op complications

**Table 3:** Risk factors associated with post pancreatectomy NODM outcomes in pancreatic adenocarcinoma

Sl. No.	Factors	Studies
1	Age	26, 30, 36, 53, 56, 58, 61
2	Gender	34, 51, 54, 59
3	Race	61
4	Family history	26, 58, 59
5	BMI	26, 34, 36, 50, 53, 56, 59, 64
6	Weight loss	42
7	Preop Hyperglycemia	23, 29, 31, 33, 36, 37, 45, 48, 50, 51, 54, 56, 59, 63
8	Preop HbA1c	23, 33, 35, 37, 44, 45, 48, 56, 63
9	Smoking	59
10	Insulin levels	22, 37, 63
11	Insulin resistance	37, 45, 47
12	C peptide	45
13	Chronic pancreatitis	27, 28, 33, 38, 62, 64
14	Tumor size	39, 40, 50
15	Tumor Staging	25
16	Dyslipidemia	38, 50
17	Deranged Liver parameters	50
18	Impaired Renal parameters (BUN, Creatinine)	50, 58
19	Operative factors	40
20	Co-morbidity	53, 62
21	Remnant pancreatic volume	21, 24, 25, 29, 31, 32, 34, 35, 36, 49, 55
22	Post-operative complications	31, 41, 43, 52, 57, 65
23	Chemotherapy	31, 44, 46
24	Radiotherapy	31

### Sociodemographic factors

Based on the narrative synthesis of risk factors associated with NODM incidence, patient age is one of the important demographic variables associated with NODM incidence. Seven (07) studies showed that older age is associated with a high incidence of NODM, compared to the younger population, which may be due to decreased β-cell regenerative capacity, higher baseline insulin resistance and high post-operative complications [23, 27, 33, 50, 53, 55, 58].

The reported gender associated high incidence of NODM varied among the studies. While three (03) studies showed a higher incidence of NODM in male patients [48, 51, 55], one study observed higher NODM incidence in female patients, possibly due to hormonal influences on insulin sensitivity [31].

Three (03) studies showed a higher NODM incidence among patients with a family history of diabetes or pancreatic cancer. A positive family history of diabetes implies a genetic predisposition to β-cell dysfunction, insulin resistance, or both. Pancreatectomy may unmask latent glucose intolerance in genetically susceptible individuals [23, 55, 56].

One included study showed the association between race and NODM incidence, which showed a higher incidence in Asian populations. Asians had higher insulin sensitivity, but lower insulin secretion reserve compared to Caucasians, leading to higher NODM risk with minimal resection [58].

### Lifestyle based factors

High pre-op BMI was associated with high NODM incidence in eight (08) studies, possibly due to increased insulin resistance, lipotoxicity and inflammation-induced β-cell dysfunction, increased ectopic fat deposition in the pancreas, exacerbating islet stress [23, 31, 33, 47, 50, 53, 56, 61].

Pre-op hyperglycemia was associated with high NODM incidence in fourteen (14) studies, implying it as one of the most important risk factors in NODM occurrence. It may be due to latent β-cell dysfunction, underlying insulin resistance, and paraneoplastic endocrine abnormalities related to pancreatic cancer [20, 26, 28, 30, 33, 34, 42, 45, 47, 48, 51, 53, 56, 60].

Elevated HbA1c was associated with high NODM incidence in nine (09) studies, as elevated HbA1c reflected subclinical glucose dysregulation or latent diabetes, making the endocrine pancreas more vulnerable post-resection [20, 30, 32, 34, 41, 42, 45, 53, 60].

One included study associated NODM incidence with weight loss, possibly due to cancer cachexia, which leads to systemic inflammation, proteolysis, and energy imbalance, which can impair insulin signalling. Weight loss >10% is linked with loss of muscle mass, including pancreatic parenchymal atrophy and reduced β-cell mass [39].

One included study showed the association between smoking and NODM incidence, as smoking is associated with β-cell dysfunction, increased insulin resistance, and chronic pancreatic inflammation [56].

### Insulin related factors

Three (03) included studies showed the association between pre-op insulin levels and NODM incidence. It may be due to low fasting insulin levels that may reflect impaired β-cell function before surgery. Postpancreatectomy, reduced pancreatic tissue may exacerbate β-cell loss, tipping borderline patients into diabetes [19, 34, 60].

Three (03) included studies showed the association between insulin resistance and NODM incidence. It may be due to insulin resistance burdens β-cells to overproduce insulin. After partial pancreatectomy, the diminished β-cell mass may be inadequate to maintain normoglycemia in insulin-resistant individuals. Tools such as HOMA-IR (Homeostasis Model Assessment-Insulin Resistance) are commonly used to quantify insulin resistance [34, 42, 44].

One included study showed the association between C-peptide levels and NODM incidence, as low C-peptide levels indicate depleted insulin-producing capacity [42].

### Chronic pancreatitis (CP)

Six (06) studies showed the association between pre-existing chronic pancreatitis and NODM incidence in pancreatic cancer patients. Due to fibrosis and inflammation, Islet cell destruction occurs, resulting in impaired insulin secretion. With glucagon and pancreatic polypeptide deficiency, worsening glucose regulation occurs. CP patients often already exhibit subclinical glucose intolerance, which is unmasked or worsened postoperatively. When the surgery further reduces the remnant pancreatic volume, it pushes marginal islet cell function past a critical threshold, precipitating NODM [24, 25, 30, 35, 59, 61].

### Tumor related factors

Three (03) included studies showed the association between tumor size and NODM incidence. Larger tumors are more likely to cause destruction of islet cells, ductal obstruction, and paraneoplastic insulin resistance. Bigger tumors often require more extensive resections, thus reducing functional endocrine tissue [36, 37, 47].

Tumor location determines the type of resection. Pancreaticoduodenectomy (Whipple’s) is indicated for pancreatic head tumors, which preserves more of the body and tail (endocrine-rich areas). Distal pancreatectomy is indicated for body/tail tumors, which removes islet-rich regions, as islet cell distribution is denser in the tail and body of the pancreas [19].

One included study showed the association between tumor stage and NODM incidence, as advanced tumor stage often reflects a more systemic inflammation, greater likelihood of pre-existing metabolic stress and requires extensive resection [22].

### Biochemical risk factors

Two (02) included studies showed the association between dyslipidemia and NODM incidence. Elevated triglycerides and low HDL are features of the metabolic syndrome, which includes insulin resistance—a predictor of poor glycemic adaptation after pancreatic resection. The inflammatory milieu associated with dyslipidemia may further worsen post-surgical glucose control [35, 47].

One included study showed the association between deranged liver parameters and NODM incidence, possibly due to liver inflammation and steatosis promoting peripheral insulin resistance [47].

Two (02) included studies showed the association between impaired renal function and NODM incidence. Chronic kidney disease (CKD) results in insulin resistance, decreased insulin clearance, and inflammatory cytokine release [47, 55].

### Operative and post-operative factors

One included study showed the association between operative factors, such as prolonged operative time and heavy blood loss, with NODM incidence. Pronged operative times may be due to complex surgical procedures, which lead to stress hyperglycemia, prolonged anesthesia effects on glucose metabolism, and tissue ischemia-reperfusion injury. Heavy blood loss leads to hemodynamic instability, hypoperfusion of the pancreatic remnant, systemic inflammatory response affecting islet cells, and insulin sensitivity [37].

Post-operative complications were associated with high NODM incidence in six (06) studies. Postoperative pancreatic fistula (POPF) and local inflammation can cause injury to islet cells in the pancreatic remnant, reducing insulin secretion. Systemic infections and sepsis induce insulin resistance via cytokine release. Delayed gastric emptying and nutritional deficits may impair glucose metabolism and insulin sensitivity. Prolonged hospitalization and recurrent complications exacerbate metabolic stress [28, 38, 40, 49, 54, 62].

### Remnant pancreatic volume

Remnant pancreatic volume was associated with high NODM incidence in eleven (11) studies, implying it as one of the most important risk factors in NODM occurrence. Partial pancreatectomy further reduces the remnant pancreatic volume, pushing marginal islet cell function past a critical threshold, precipitating NODM [18, 21, 22, 26, 28, 29, 31, 32, 33, 46, 52].

### Co-morbidity

Two (02) included studies showed the association between pre-op co-morbidity and NODM incidence. High Charlson co-morbidity index (CCI) scores are associated with impaired glucose homeostasis, increased baseline insulin resistance (e.g., due to obesity, cardiovascular disease, CKD). These factors can amplify the glycemic impact of pancreatectomy, especially in major resections [50, 59].

### Chemotherapy

Three (03) studies showed the association between chemotherapy and NODM incidence. Some chemotherapy agents cause direct pancreatic toxicity, while some other agents may damage pancreatic islet cells, impairing insulin secretion. Chemotherapy induces systemic inflammation with cytokine release, promoting insulin resistance. Chemotherapy-associated anorexia and malabsorption affect glucose homeostasis. Chemotherapeutic drugs may also interfere with insulin receptor signaling [28, 41, 43].

### Radiotherapy

One included study showed the association between radiotherapy and NODM incidence. Radiotherapy may cause direct islet cell damage, which may reduce insulin secretion. Fibrosis and vascular injury in the pancreatic remnant impair blood supply and endocrine function. The inflammatory response triggered by radiation may promote insulin resistance [28].

The forest plot of the associated risk factors with the strongest weight of evidence was done and included in the Supplementary File 2.

## DISCUSSION

To the best of our understanding, this is the first systematic review examining the incidence of NODM and its related risk factors specifically in patients with Pancreatic Adenocarcinoma (PAC) who have undergone partial pancreatectomy. Our analysis incorporated research from both developed and developing countries, comprising a total of 45 studies.

Among the patients in this systematic review who underwent partial pancreatectomy for pancreatic adenocarcinoma, the overall incidence of NODM in the studied population was 24.5%, affecting up to one-fourth of the study population. In the 2 subgroups according to the type of resection (PD or DP) done for pancreatic cancer cases, patients who underwent PD, the incidence of NODM was 23.2%, and for DP resection cases, the NODM incidence was 26.3%.

The occurrence of NODM has been evaluated concerning various types of pancreatic surgeries in prior studies. Multiple clinical studies produced inconsistent outcomes. While some researchers suggested that patients undergoing DP faced an increased risk of developing NODM, other studies indicated a marginally higher incidence of NODM in patients who had DP compared to those who underwent PD, yet this difference was not statistically significant [46, 63]. Our review mirrored these findings, revealing that the incidence of NODM after DP (26.3%) was slightly above that of PD (23.2%).

A systematic review and meta-analysis were undertaken by Beger et al, in post PD resections for both benign and malignant tumors, which included quantitative assessments from 19 studies. They found that the cumulative incidence of NODM was 14.5% for the group with benign tumors, 15.5% for the group with malignant tumors, and 22.2% for the combined group of both benign and malignant tumors [64].

A similar systematic review and Meta-analysis were undertaken by De Bruijn et al, in exclusively post DP groups for pancreatic disorders involving quantitative analysis of 26 studies. This review indicates that the average cumulative incidence of NODM) following DP procedures conducted for benign or potentially malignant conditions is 14% [65]. But a recent study by Yu et al., who did a systematic review and Meta-analysis of NODM after DP for pancreatic disorders involving quantitative analysis of 18 studies. The rate of NODM was 23% in patients with pancreatic tumors. However, the study included both benign and malignant lesions [66]. Our study included only malignant pancreatic adenocarcinoma cases and showed slightly higher NODM incidence for both PD and DP types.

Regarding the risk factors associated with NODM incidence in this group of patients, we did a narrative synthesis of up to 37 different variables related to NODM incidence. Out of all these variables, older age, high BMI, pre-op hyperglycemia, pre-op high HbA1c, pre-existing chronic pancreatitis, poor remnant pancreatic volume, and severe post-operative complications were associated with NODM incidence.

These results were consistent with those previously published studies and reviews.

Older patients undergoing pancreatectomy for PAC were associated with high NODM incidence. A similar finding was observed in a study, which showed that age >60 years was an independent risk factor for NODM after pancreaticoduodenectomy [67]. Regarding gender, the results were mixed. Some studies suggest females were more susceptible, possibly due to hormonal influences on insulin sensitivity [31]. Some included studies suggest where males are more likely to develop chronic pancreatitis and thus more prone to developing DM [68]. Others find no significant difference in relation to gender, after adjusting for BMI and age [69].

Regarding race and ethnicity as influencers of NODM risk, our analysis showed a higher incidence in Asian populations [58], which was similar to reported findings by a study that indicated that being from a non-White race or ethnicity was independently linked to poorer outcomes following pancreatic cancer surgery [70]. Another study observed that a higher baseline prevalence of pre-diabetes and NODM in the Indian cohort compared to Western reports [71]. Regarding the Family history of diabetes, as an influencer of NODM risk, our analysis showed higher NODM incidence in this population. One study reflected the similar findings, which observed that Family history of diabetes emerged as a significant non-modifiable risk factor, especially in patients without pre-existing hyperglycemia [72].

Our analysis showed that high BMI was associated with NODM incidence in this population. A study concurred with our findings, which showed that high BMI had both poorer glycemic profiles and a greater tendency to develop NODM after surgery [73].

Our analysis showed that Pre-op hyperglycemia and high HbA1c were among the most important risk factors associated with NODM incidence. Similar findings were observed in a study that showed that Preoperative impaired fasting glucose and mildly elevated HbA1c predicted postoperative diabetes [74]. Another study showed that patients with elevated preoperative HbA1c had a significantly higher risk of developing NODM postoperatively [75].

In our analysis, pre-existing chronic pancreatitis as a risk factor was associated with NODM incidence.

One study showed similar findings that patients with pre-existing CP had significantly lower insulin and C-peptide levels of post-surgery, predisposing them to NODM [76]. Another study showed that Chronic inflammation and fibrosis reduce the functional β-cell mass, making patients prone to NODM post pancreatectomy [77]. Another study indicates that it is conceivable that chronic pancreatitis may affect the likelihood of developing NODM [15].

Regarding the tumor size, as an influencer of NODM risk, our analysis showed higher NODM incidence in this population. One study showed similar findings that a tumor size was independently associated with increased NODM incidence post-resection in localized disease [78].

Our analysis showed that remnant pancreatic volume was one of the most important risk factors associated with NODM incidence. One recent study showed similar findings that reduced remnant pancreatic volume, causing beta-cell dysfunction, might be one of the mechanisms of NODM secondary to PAC [79]. A different study noted that it is likely that the volume and health of remaining pancreatic tissue may affect the likelihood of developing new-onset diabetes mellitus [15].

Our analysis showed that a high incidence of post-operative complications was associated with NODM incidence. Few studies showed similar findings that patients with clinically relevant postoperative pancreatic fistula had a significantly higher incidence of NODM at 6 months post-pancreatectomy [80, 81]. Another study showed that patients with delayed gastric emptying had impaired glycemic control at 3 and 6 months postoperatively [82].

### Limitations

It is important to acknowledge that this review has certain limitations and should be interpreted accordingly. To begin with, since the review was limited to works published in English, there is a possibility that relevant studies in other languages may have been missed. Secondly, variations in surgical methods, such as the amount of pancreas removed during routine clinical practices, can differ significantly between institutions, surgeons, and patients, along with surgical techniques like laparotomy or laparoscopy, and whether the spleen was preserved, leading to substantial heterogeneity in the surgical approaches. Third, studies employed different methods for reporting the incidence of NODM, which lacked standardization concerning follow-up periods. Fourth, the risk stratification for pre-existing pancreatic conditions could not be thoroughly clarified, nor were confounding factors adequately addressed, as this was based on the original designs and reports of the studies included. Fifth, potential for publication bias, as smaller studies or those reporting null associations between surgical or metabolic factors and NODM may be underrepresented in the literature. This selective reporting can overestimate pooled incidence or effect sizes, thereby limiting the generalizability and external validity of the analysis.

## MATERIALS AND METHODS

We submitted our review protocol and registered with PROSPERO (CRD420251024206) and followed the established protocol for systematic reviews as per PRISMA statement [83].

### Search strategy

We conducted searches in the databases MEDLINE, Cochrane, Embase, and Scopus to identify relevant studies, following the guidelines outlined in the Peer Review of Electronic Search Strategies (PRESS) [84]. The PubMed database was queried using medical subject headings (MeSH). Search terms included “Pancreatic carcinoma,” “Pancreatectomy,” and “Diabetes mellitus.” Our search strategy was broad to ensure a comprehensive review of the available literature and to capture all pertinent evidence. We also refined our search strategy by closely reviewing the references of the articles we gathered. Our search was confined to English-language publications published from January 2000 to March 2025 (Supplementary File 3).

### Eligibility criteria and study selection

The research selection process involved two reviewers (KAK & AKR). All studies that provided information on postoperative pancreatic endocrine function in patients with pancreatic adenocarcinoma (PAC) who had either a Pancreaticoduodenectomy (PD) or Distal Pancreatectomy (DP) were considered in the initial evaluation. Studies that (1) did not report the incidence of preoperative diabetes, (2) concentrated on surgical techniques, and (3) had a follow-up period of less than 3 months were excluded from the analysis.

### Data extraction and quality assessment

#### Data extraction

Two investigators (KAK & AKR) conducted a separate screening of the titles, abstracts, and full articles. The extracted data included the patients’ gender, average age, existence of preoperative diabetes; occurrence of postoperative diabetes; incidence of new onset diabetes mellitus (NODM), the type of pancreatectomy performed (PD or DP), as well as the criteria used to diagnose diabetes, which included fasting blood glucose (FBG), glycated hemoglobin (HbA1c) levels, or an oral glucose tolerance test (OGTT); and the duration of the follow-up period.

A narrative synthesis outlines the risk factors linked to the onset of NODM in these patient populations, providing an analysis by subgroups in relation to PD versus DP groups.

### Quality assessment

Quality assessments were conducted to assess the robustness and caliber of the evidence produced by the studies. The Newcastle–Ottawa scale (NOS) was utilized to evaluate all cohort studies, with a NOS score of 6 or higher indicating a study of high quality [85].

### Statistical analyses

The overall incidence of NODM was determined using the incidence rate method. When the publication did not provide the NODM rate, it was computed by dividing the number of patients with NODM by the number of patients who did not have preoperative diabetes. Subgroup analyses were conducted on the groups of patients with surgical procedures of PD vs. DP. A narrative synthesis was performed to assess the associated risk factors.

## CONCLUSIONS

This systematic review aims to remind surgeons that a deficiency in pancreatic endocrine function is a significant long-term complication following pancreatectomy. Older age, high BMI, pre-op hyperglycemia, pre-op high HbA1c, pre-existing chronic pancreatitis, poor remnant pancreatic volume, and severe post-operative complications were associated with NODM incidence. Larger cohort studies with more extensive patient populations should be carried out to better understand the risk factors linked to NODM following pancreatectomy. It is essential to implement suitable screening and enhance patient education for individuals with recognized risk factors.

## SUPPLEMENTARY MATERIALS



## Abbreviations

PAC: Pancreatic Adenocarcinoma
NODM: New-Onset Diabetes Mellitus
PD: Pancreaticoduodenectomy
DP: Distal Pancreatectomy
TP: Total Pancreatectomy
BMI: Body Mass Index
OGTT: Oral Glucose Tolerance Test
HbA1c: Glycated Hemoglobin
CP: Chronic Pancreatitis
POPF: Postoperative Pancreatic Fistula
BMI: Body Mass Index

## References

[1] Sung H, Ferlay J, Siegel RL, Laversanne M, Soerjomataram I, Jemal A, Bray F. Global Cancer Statistics 2020: GLOBOCAN Estimates of Incidence and Mortality Worldwide for 36 Cancers in 185 Countries. CA Cancer J Clin. 2021; 71:209–49. 10.3322/caac.21660. 33538338

[2] Becker AE, Hernandez YG, Frucht H, Lucas AL. Pancreatic ductal adenocarcinoma: risk factors, screening, and early detection. World J Gastroenterol. 2014; 20:11182–98. 10.3748/wjg.v20.i32.11182. 25170203 PMC4145757

[3] Wang F, Gupta S, Holly EA. Diabetes mellitus and pancreatic cancer in a population-based case-control study in the San Francisco Bay Area, California. Cancer Epidemiol Biomarkers Prev. 2006; 15:1458–63. 10.1158/1055-9965.EPI-06-0188. 16896032

[4] Conlon KC, Klimstra DS, Brennan MF. Long-term survival after curative resection for pancreatic ductal adenocarcinoma. Clinicopathologic analysis of 5-year survivors. Ann Surg. 1996; 223:273–79. 10.1097/00000658-199603000-00007. 8604907 PMC1235115

[5] Lahat G, Ben Haim M, Nachmany I, Sever R, Blachar A, Nakache R, Klausner JM. Pancreatic incidentalomas: high rate of potentially malignant tumors. J Am Coll Surg. 2009; 209:313–19. 10.1016/j.jamcollsurg.2009.05.009. 19717035

[6] Lemmens VE, Bosscha K, van der Schelling G, Brenninkmeijer S, Coebergh JW, de Hingh IH. Improving outcome for patients with pancreatic cancer through centralization. Br J Surg. 2011; 98:1455–62. 10.1002/bjs.7581. 21717423

[7] Donahue TR, Reber HA. Pancreatic surgery. Curr Opin Gastroenterol. 2013; 29:552–58. 10.1097/MOG.0b013e3283639359. 23892537

[8] Bliss LA, Yang CJ, Chau Z, Ng SC, McFadden DW, Kent TS, Moser AJ, Callery MP, Tseng JF. Patient selection and the volume effect in pancreatic surgery: unequal benefits? HPB (Oxford). 2014; 16:899–906. 10.1111/hpb.12283. 24905343 PMC4238856

[9] Ferrone CR, Pieretti-Vanmarcke R, Bloom JP, Zheng H, Szymonifka J, Wargo JA, Thayer SP, Lauwers GY, Deshpande V, Mino-Kenudson M, Fernández-del Castillo C, Lillemoe KD, Warshaw AL. Pancreatic ductal adenocarcinoma: long-term survival does not equal cure. Surgery. 2012 (Suppl 1); 152:S43–49. 10.1016/j.surg.2012.05.020. 22763261 PMC3806092

[10] Wu L, Nahm CB, Jamieson NB, Samra J, Clifton-Bligh R, Mittal A, Tsang V. Risk factors for development of diabetes mellitus (Type 3c) after partial pancreatectomy: A systematic review. Clin Endocrinol (Oxf). 2020; 92:396–406. 10.1111/cen.14168. 32017157

[11] American Diabetes Association Professional Practice Committee. 2. Diagnosis and Classification of Diabetes: Standards of Care in Diabetes-2024. Diabetes Care. 2024 (Suppl 1); 47:S20–42. 10.2337/dc24-S002. 38078589 PMC10725812

[12] Shingyoji A, Mikata R, Ogasawara S, Kusakabe Y, Yasui S, Sugiyama H, Ohno I, Kato J, Takano S, Yoshitomi H, Ohtsuka M, Kato N. Diverse transitions in diabetes status during the clinical course of patients with resectable pancreatic cancer. Jpn J Clin Oncol. 2020; 50:1403–11. 10.1093/jjco/hyaa136. 32761096

[13] Pelaez-Luna M, Takahashi N, Fletcher JG, Chari ST. Resectability of presymptomatic pancreatic cancer and its relationship to onset of diabetes: a retrospective review of CT scans and fasting glucose values prior to diagnosis. Am J Gastroenterol. 2007; 102:2157–63. 10.1111/j.1572-0241.2007.01480.x. 17897335

[14] Schrader H, Menge BA, Breuer TG, Ritter PR, Uhl W, Schmidt WE, Holst JJ, Meier JJ. Impaired glucose-induced glucagon suppression after partial pancreatectomy. J Clin Endocrinol Metab. 2009; 94:2857–63. 10.1210/jc.2009-0826. 19491219

[15] Henkel E, Menschikowski M, Koehler C, Leonhardt W, Hanefeld M. Impact of glucagon response on postprandial hyperglycemia in men with impaired glucose tolerance and type 2 diabetes mellitus. Metabolism. 2005; 54:1168–73. 10.1016/j.metabol.2005.03.024. 16125528

[16] Muscogiuri G, Mezza T, Prioletta A, Sorice GP, Clemente G, Sarno G, Nuzzo G, Pontecorvi A, Holst JJ, Giaccari A. Removal of duodenum elicits GLP-1 secretion. Diabetes Care. 2013; 36:1641–46. 10.2337/dc12-0811. 23393218 PMC3661831

[17] Barone BB, Yeh HC, Snyder CF, Peairs KS, Stein KB, Derr RL, Wolff AC, Brancati FL. Postoperative mortality in cancer patients with preexisting diabetes: systematic review and meta-analysis. Diabetes Care. 2010; 33:931–39. 10.2337/dc09-1721. 20351229 PMC2845055

[18] Kanwat S, Singh H, Sharma AK, Sharma V, Gupta P, Gupta V, Yadav TD, Gupta R. Pancreatic Dysfunction and Reduction in Quality of Life Is Common After Pancreaticoduodenectomy. Dig Dis Sci. 2023; 68:3167–73. 10.1007/s10620-023-07966-6. 37160540

[19] Niwano F, Babaya N, Hiromine Y, Matsumoto I, Kamei K, Taketomo Y, Yoshida S, Takeyama Y, Noso S, Ikegami H. Three-Year Observation of Glucose Metabolism After Pancreaticoduodenectomy: A Single-Center Prospective Study in Japan. J Clin Endocrinol Metab. 2022; 107:3362–69. 10.1210/clinem/dgac529. 36074913 PMC9693916

[20] Maxwell DW, Jajja MR, Tariq M, Mahmooth Z, Galindo RJ, Sweeney JF, Sarmiento JM. Development of Diabetes after Pancreaticoduodenectomy: Results of a 10-Year Series Using Prospective Endocrine Evaluation. J Am Coll Surg. 2019; 228:400–12.e2. 10.1016/j.jamcollsurg.2018.12.042. 30690075

[21] Singh AN, Pal S, Kilambi R, Madhusudhan KS, Dash NR, Tandon N, Sahni P. Diabetes after pancreaticoduodenectomy: can we predict it? J Surg Res. 2018; 227:211–19. 10.1016/j.jss.2018.02.010. 29804855

[22] Yun SP, Seo HI, Kim S, Kim DU, Baek DH. Does the pancreatic volume reduction rate using serial computed tomographic volumetry predict new onset diabetes after pancreaticoduodenectomy? Medicine (Baltimore). 2017; 96:e6491. 10.1097/MD.0000000000006491. 28353594 PMC5380278

[23] Pannala R, Leirness JB, Bamlet WR, Basu A, Petersen GM, Chari ST. Prevalence and clinical profile of pancreatic cancer-associated diabetes mellitus. Gastroenterology. 2008; 134:981–87. 10.1053/j.gastro.2008.01.039. 18395079 PMC2323514

[24] Wu JM, Ho TW, Kuo TC, Yang CY, Lai HS, Chiang PY, Hsieh SH, Lai F, Tien YW. Glycemic Change After Pancreaticoduodenectomy: A Population-Based Study. Medicine (Baltimore). 2015; 94:e1109. 10.1097/MD.0000000000001109. 26166104 PMC4504605

[25] Oh HM, Yoon YS, Han HS, Kim JH, Cho JY, Hwang DW. Risk factors for pancreatogenic diabetes after pancreaticoduodenectomy. Korean J Hepatobiliary Pancreat Surg. 2012; 16:167–71. 10.14701/kjhbps.2012.16.4.167. 26388929 PMC4575001

[26] Ferrara MJ, Lohse C, Kudva YC, Farnell MB, Que FG, Reid-Lombardo KM, Donohue JH, Nagorney DM, Chari ST, Vege SS, Kendrick ML. Immediate post-resection diabetes mellitus after pancreaticoduodenectomy: incidence and risk factors. HPB (Oxford). 2013; 15:170–74. 10.1111/j.1477-2574.2012.00520.x. 23374356 PMC3572276

[27] Bock EA, Hurtuk MG, Shoup M, Aranha GV. Late complications after pancreaticoduodenectomy with pancreaticogastrostomy. J Gastrointest Surg. 2012; 16:914–19. 10.1007/s11605-011-1805-2. 22374385

[28] You DD, Choi SH, Choi DW, Heo JS, Ho CY, Kim WS. Long-term effects of pancreaticoduodenectomy on glucose metabolism. ANZ J Surg. 2012; 82:447–51. 10.1111/j.1445-2197.2012.06080.x. 22571457

[29] Imamura S, Niwano F, Babaya N, Hiromine Y, Matsumoto I, Kamei K, Yoshida Y, Taketomo Y, Yoshida S, Takeyama Y, Noso S, Maeda N, Ikegami H. High Incidence of Diabetes Mellitus After Distal Pancreatectomy and Its Predictors: A Long-term Follow-up Study. J Clin Endocrinol Metab. 2024; 109:619–30. 10.1210/clinem/dgad634. 37889837 PMC10876401

[30] Tariq M, Jajja MR, Maxwell DW, Galindo RJ, Sweeney JF, Sarmiento JM. Diabetes development after distal pancreatectomy: results of a 10-year series. HPB (Oxford). 2020; 22:1034–41. 10.1016/j.hpb.2019.10.2440. 31718897

[31] Kang JS, Jang JY, Kang MJ, Kim E, Jung W, Chang J, Shin Y, Han Y, Kim SW. Endocrine Function Impairment After Distal Pancreatectomy: Incidence and Related Factors. World J Surg. 2016; 40:440–46. 10.1007/s00268-015-3228-9. 26330237

[32] Shirakawa S, Matsumoto I, Toyama H, Shinzeki M, Ajiki T, Fukumoto T, Ku Y. Pancreatic volumetric assessment as a predictor of new-onset diabetes following distal pancreatectomy. J Gastrointest Surg. 2012; 16:2212–19. 10.1007/s11605-012-2039-7. 23054900 PMC3508270

[33] Shen J, Cao J, He J, Yu H, Chen M. Clinical utility of resected pancreatic volume ratio calculation for predicting postoperative new-onset diabetes mellitus after distal pancreatectomy-a propensity-matched analysis. Heliyon. 2023; 9:e15998. 10.1016/j.heliyon.2023.e15998. 37206003 PMC10189175

[34] Izumo W, Higuchi R, Yazawa T, Uemura S, Shiihara M, Yamamoto M. Evaluation of allowable pancreatic resection rate depending on preoperative risk factors for new-onset diabetes mellitus after distal pancreatectomy. Pancreatology. 2020; 20:1526–33. 10.1016/j.pan.2020.08.005. 32855059

[35] Wu JM, Ho TW, Yang CY, Lee PH, Tien YW. Changes in glucose metabolism after distal pancreatectomy: a nationwide database study. Oncotarget. 2018; 9:11100–108. 10.18632/oncotarget.24325. 29541399 PMC5834261

[36] Kwon W, Jang JY, Kim JH, Chang YR, Jung W, Kang MJ, Kim SW. An Analysis of Complications, Quality of Life, and Nutritional Index After Laparoscopic Distal Pancreatectomy with Regard to Spleen Preservation. J Laparoendosc Adv Surg Tech A. 2016; 26:335–42. 10.1089/lap.2015.0171. 26982249

[37] Malleo G, Damoli I, Marchegiani G, Esposito A, Marchese T, Salvia R, Bassi C, Butturini G. Laparoscopic distal pancreatectomy: analysis of trends in surgical techniques, patient selection, and outcomes. Surg Endosc. 2015; 29:1952–62. 10.1007/s00464-014-3890-2. 25303912

[38] Stutchfield BM, Joseph S, Duckworth AD, Garden OJ, Parks RW. Distal pancreatectomy: what is the standard for laparoscopic surgery? HPB (Oxford). 2009; 11:210–14. 10.1111/j.1477-2574.2009.00008.x. 19590649 PMC2697890

[39] Chari ST, Leibson CL, Rabe KG, Timmons LJ, Ransom J, de Andrade M, Petersen GM. Pancreatic cancer-associated diabetes mellitus: prevalence and temporal association with diagnosis of cancer. Gastroenterology. 2008; 134:95–101. 10.1053/j.gastro.2007.10.040. 18061176 PMC2271041

[40] Adam U, Makowiec F, Riediger H, Trzeczak S, Benz S, Hopt UT. Distale Pankreasresektion--Indikation, Verfahren, postoperative Ergebnisse [Distal pancreatic resection--indications, techniques and complications]. Zentralbl Chir. 2001; 126:908–12. 10.1055/s-2001-19149. 11753802

[41] Lee JS, Sohn M, Kim K, Yoon YS, Lim S. Glucose Regulation after Partial Pancreatectomy: A Comparison of Pancreaticoduodenectomy and Distal Pancreatectomy in the Short and Long Term. Diabetes Metab J. 2023; 47:703–14. 10.4093/dmj.2022.0205. 37349082 PMC10555545

[42] Ishida J, Toyama H, Matsumoto I, Shirakawa S, Terai S, Yamashita H, Yanagimoto H, Asari S, Kido M, Fukumoto T. Glucose Tolerance after Pancreatectomy: A Prospective Observational Follow-Up Study of Pancreaticoduodenectomy and Distal Pancreatectomy. J Am Coll Surg. 2021; 233:753–62. 10.1016/j.jamcollsurg.2021.08.688. 34530126

[43] Shaw K, Thomas AS, Rosario V, Kwon W, Schrope BA, Sugahara K, Chabot JA, Genkinger JM, Kluger MD. Long term quality of life amongst pancreatectomy patients with diabetes mellitus. Pancreatology. 2021; 21:501–508. 10.1016/j.pan.2021.01.012. 33509685

[44] Niwano F, Babaya N, Hiromine Y, Matsumoto I, Kamei K, Noso S, Taketomo Y, Takeyama Y, Kawabata Y, Ikegami H. Glucose Metabolism After Pancreatectomy: Opposite Extremes Between Pancreaticoduodenectomy and Distal Pancreatectomy. J Clin Endocrinol Metab. 2021; 106:e2203–14. 10.1210/clinem/dgab036. 33484558 PMC8063252

[45] Maxwell DW, Jajja MR, Galindo RJ, Zhang C, Nadeem SO, Sweeney JF, Blair CM, Sarmiento JM. Post-Pancreatectomy Diabetes Index: A Validated Score Predicting Diabetes Development after Major Pancreatectomy. J Am Coll Surg. 2020; 230:393–402.e3. 10.1016/j.jamcollsurg.2019.12.016. 31981618

[46] Maignan A, Ouaïssi M, Turrini O, Regenet N, Loundou A, Louis G, Moutardier V, Dahan L, Pirrò N, Sastre B, Delpero JR, Sielezneff I. Risk factors of exocrine and endocrine pancreatic insufficiency after pancreatic resection: A multi-center prospective study. J Visc Surg. 2018; 155:173–81. 10.1016/j.jviscsurg.2017.10.007. 29396112

[47] Wang S, Zhou H, Cai K, Fan Y, Yang X, Zhang B, Wu Y. Predictive value of perioperative fasting blood glucose for post pancreatectomy diabetes mellitus in pancreatic ductal carcinoma patients. World J Surg Oncol. 2025; 23:55. 10.1186/s12957-025-03705-5. 39955538 PMC11830169

[48] Yoo D, Kang M, Jung J. Risk of Ischemic Heart Disease in Patients With Postpancreatectomy Diabetes and Pancreatic Cancer: A Population-Based Study. J Am Heart Assoc. 2023; 12:e031321. 10.1161/JAHA.123.031321. 38084734 PMC10863790

[49] Schranz A, Sternad C, Aziz F, Wagner D, Kornprat P, Sucher R, Jost PJ, Wölfler A, Pieber TR, Sourij H, Riedl JM, Aberer F. Incidence of Diabetes Mellitus and Its Impact on Outcomes in Patients Undergoing Surgical Pancreatectomy for Non-Malignant and Malignant Pancreatobiliary Diseases-A Retrospective Analysis. J Clin Med. 2023; 12:7532. 10.3390/jcm12247532. 38137600 PMC10744322

[50] Thomas AS, Huang Y, Kwon W, Schrope BA, Sugahara K, Chabot JA, Wright JD, Kluger MD. Prevalence and Risk Factors for Pancreatic Insufficiency After Partial Pancreatectomy. J Gastrointest Surg. 2022; 26:1425–35. 10.1007/s11605-022-05302-3. 35318597

[51] Hamad A, Hyer JM, Thayaparan V, Salahuddin A, Cloyd JM, Pawlik TM, Ejaz A. Pancreatogenic Diabetes after Partial Pancreatectomy: A Common and Understudied Cause of Morbidity. J Am Coll Surg. 2022; 235:838–45. 10.1097/XCS.0000000000000360. 36102556

[52] Yamada D, Takahashi H, Asukai K, Hasegawa S, Wada H, Matsuda C, Yasui M, Omori T, Miyata H, Sakon M. Investigation of the influence of pancreatic surgery on new-onset and persistent diabetes mellitus. Ann Gastroenterol Surg. 2021; 5:575–84. 10.1002/ags3.12435. 34337306 PMC8316753

[53] Yamamoto-Kataoka S, Shimizu S, Yamazaki H, Murakami K, Nishizaki D, Fukuhara S, Inagaki N, Yamamoto Y. Development of a preoperative prediction model for new-onset diabetes mellitus after partial pancreatectomy: A retrospective cohort study. Medicine (Baltimore). 2021; 100:e26311. 10.1097/MD.0000000000026311. 34128870 PMC8213311

[54] Mayeux SE, Kwon W, Rosario VL, Rossmer I, Schrope BA, Chabot JA, Kluger MD. Long-term health after pancreatic surgery: the view from 9.5 years. HPB (Oxford). 2021; 23:595–600. 10.1016/j.hpb.2020.08.017. 32988751

[55] Lee CYC, Depczynski B, Poynten A, Haghighi KS. Diabetes-related outcomes after pancreatic surgery. ANZ J Surg. 2020; 90:2004–10. 10.1111/ans.16129. 32691521

[56] Kusakabe J, Anderson B, Liu J, Williams GA, Chapman WC, Doyle MMB, Khan AS, Sanford DE, Hammill CW, Strasberg SM, Hawkins WG, Fields RC. Long-Term Endocrine and Exocrine Insufficiency After Pancreatectomy. J Gastrointest Surg. 2019; 23:1604–13. 10.1007/s11605-018-04084-x. 30671791 PMC6646099

[57] Karlin NJ, Amin SB, Kosiorek HE, Buras MR, Verona PM, Cook CB. Survival and glycemic control outcomes among patients with coexisting pancreatic cancer and diabetes mellitus. Future Sci OA. 2018; 4:FSO291. 10.4155/fsoa-2017-0144. 29682326 PMC5905610

[58] Nguyen A, Demirjian A, Yamamoto M, Hollenbach K, Imagawa DK. Development of Postoperative Diabetes Mellitus in Patients Undergoing Distal Pancreatectomy *versus* Whipple Procedure. Am Surg. 2017; 83:1050–53. 29391093

[59] Elliott IA, Epelboym I, Winner M, Allendorf JD, Haigh PI. Population-Level Incidence and Predictors of Surgically Induced Diabetes and Exocrine Insufficiency after Partial Pancreatic Resection. Perm J. 2017; 21:16–95. 10.7812/TPP/16-095. 28406793 PMC5391783

[60] Burkhart RA, Gerber SM, Tholey RM, Lamb KM, Somasundaram A, McIntyre CA, Fradkin EC, Ashok AP, Felte RF, Mehta JM, Rosato EL, Lavu H, Jabbour SA, et al. Incidence and severity of pancreatogenic diabetes after pancreatic resection. J Gastrointest Surg. 2015; 19:217–25. 10.1007/s11605-014-2669-z. 25316483

[61] Hirata K, Nakata B, Amano R, Yamazoe S, Kimura K, Hirakawa K. Predictive factors for change of diabetes mellitus status after pancreatectomy in preoperative diabetic and nondiabetic patients. J Gastrointest Surg. 2014; 18:1597–603. 10.1007/s11605-014-2521-5. 25002020

[62] White MA, Agle SC, Fuhr HM, Mehaffey JH, Waibel BH, Zervos EE. Impact of pancreatic cancer and subsequent resection on glycemic control in diabetic and nondiabetic patients. Am Surg. 2011; 77:1032–37. 21944519

[63] Mori Y, Ohtsuka T, Tsutsumi K, Yasui T, Ueda J, Takahata S, Nakamura M, Tanaka M. Different incretin responses after pancreatoduodenectomy and distal pancreatectomy. Pancreas. 2012; 41:455–60. 10.1097/MPA.0b013e3182319d7c. 22422137

[64] Beger HG, Poch B, Mayer B, Siech M. New Onset of Diabetes and Pancreatic Exocrine Insufficiency After Pancreaticoduodenectomy for Benign and Malignant Tumors: A Systematic Review and Meta-analysis of Long-term Results. Ann Surg. 2018; 267:259–70. 10.1097/SLA.0000000000002422. 28834847

[65] De Bruijn KM, van Eijck CH. New-onset diabetes after distal pancreatectomy: a systematic review. Ann Surg. 2015; 261:854–61. 10.1097/SLA.0000000000000819. 24983994

[66] Yu J, Sun R, Han X, Liu Z. New-Onset Diabetes Mellitus After Distal Pancreatectomy: A Systematic Review and Meta-Analysis. J Laparoendosc Adv Surg Tech A. 2020; 30:1215–22. 10.1089/lap.2020.0090. 32559393

[67] Park HM, Park SJ, Han SS, Kim SH. Surgery for elderly patients with resectable pancreatic cancer, a comparison with non-surgical treatments: a retrospective study outcomes of resectable pancreatic cancer. BMC Cancer. 2019; 19:1090. 10.1186/s12885-019-6255-3. 31718565 PMC6852721

[68] Dumitrascu T, Eftimie M, Aiordachioae A, Stroescu C, Dima S, Ionescu M, Popescu I. Male gender and increased body mass index independently predicts clinically relevant morbidity after spleen-preserving distal pancreatectomy. World J Gastrointest Surg. 2018; 10:84–89. 10.4240/wjgs.v10.i8.84. 30510633 PMC6259023

[69] Zhou Y, Drake J, Deneve JL, Behrman SW, Dickson PV, Shibata D, Glazer ES. Rising BMI Is Associated with Increased Rate of Clinically Relevant Pancreatic Fistula after Distal Pancreatectomy for Pancreatic Adenocarcinoma. Am Surg. 2019; 85:1376–80. 31908221

[70] Alwatari Y, Mosquera CM, Khoraki J, Rustom S, Wall N, Sevdalis AE, Stover W, Trevino JG, Kaplan B. The impact of race/ethnicity on pancreaticoduodenectomy outcomes for pancreatic cancer. J Surg Oncol. 2023; 127:99–108. 10.1002/jso.27113. 36177773 PMC10092121

[71] Shrikhande SV, Barreto SG, Somashekar BA, Suradkar K, Shetty GS, Talole S, Sirohi B, Goel M, Shukla PJ. Evolution of pancreatoduodenectomy in a tertiary cancer center in India: improved results from service reconfiguration. Pancreatology. 2013; 13:63–71. 10.1016/j.pan.2012.11.302. 23395572

[72] Ferrone CR, Marchegiani G, Hong TS, Ryan DP, Deshpande V, McDonnell EI, Sabbatino F, Santos DD, Allen JN, Blaszkowsky LS, Clark JW, Faris JE, Goyal L, et al. Radiological and surgical implications of neoadjuvant treatment with FOLFIRINOX for locally advanced and borderline resectable pancreatic cancer. Ann Surg. 2015; 261:12–17. 10.1097/SLA.0000000000000867. 25599322 PMC4349683

[73] Dai M, Xing C, Shi N, Wang S, Wu G, Liao Q, Zhang T, Chen G, Wu W, Guo J, Liu Z. Risk factors for new-onset diabetes mellitus after distal pancreatectomy. BMJ Open Diabetes Res Care. 2020; 8:e001778. 10.1136/bmjdrc-2020-001778. 33122295 PMC7597507

[74] Hashimoto D, Chikamoto A, Taki K, Arima K, Yamashita Y, Ohmuraya M, Hirota M, Baba H. Residual total pancreatectomy: Short-and long-term outcomes. Pancreatology. 2016; 16:646–51. 10.1016/j.pan.2016.04.034. 27189919

[75] Kunstman JW, Healy JM, Araya DA, Salem RR. Effects of preoperative long-term glycemic control on operative outcomes following pancreaticoduodenectomy. Am J Surg. 2015; 209:1053–62. 10.1016/j.amjsurg.2014.06.029. 25242683

[76] Kleeff J, Whitcomb DC, Shimosegawa T, Esposito I, Lerch MM, Gress T, Mayerle J, Drewes AM, Rebours V, Akisik F, Muñoz JED, Neoptolemos JP. Chronic pancreatitis. Nat Rev Dis Primers. 2017; 3:17060. 10.1038/nrdp.2017.60. 28880010

[77] Meier JJ, Giese A. Diabetes associated with pancreatic diseases. Curr Opin Gastroenterol. 2015; 31:400–6. 10.1097/MOG.0000000000000199. 26125315

[78] Ansari D, Bauden M, Bergström S, Rylance R, Marko-Varga G, Andersson R. Relationship between tumour size and outcome in pancreatic ductal adenocarcinoma. Br J Surg. 2017; 104:600–7. 10.1002/bjs.10471. 28177521

[79] Yang J, Tan C, Liu Y, Zheng Z, Liu X, Chen Y. Remnant Pancreas Volume Affects New-Onset Impaired Glucose Homeostasis Secondary to Pancreatic Cancer. Biomedicines. 2024; 12:1653. 10.3390/biomedicines12081653. 39200119 PMC11351567

[80] Chang JH, Kakati RT, Wehrle C, Naples R, Joyce D, Augustin T, Simon R, Walsh RM, Dahdaleh FS, Spanheimer P, Salti I, Parente A, Naffouje SA. Incidence of clinically relevant postoperative pancreatic fistula in patients undergoing open and minimally invasive pancreatoduodenectomy: a population-based study. J Minim Invasive Surg. 2024; 27:95–108. 10.7602/jmis.2024.27.2.95. 38887001 PMC11187613

[81] Kumar S, Chandra A, Madhavan SM, Kumar D, Chauhan S, Pandey A, Masood S. Predictors and Outcomes of Pancreatic Fistula Following Pancreaticoduodenectomy: a Dual Center Experience. Indian J Surg Oncol. 2021; 12:22–30. 10.1007/s13193-020-01195-3. 33814828 PMC7960792

[82] Dominguez OH, Grigorian A, Wolf RF, Imagawa DK, Nahmias JT, Jutric Z. Delayed gastric emptying is associated with increased risk of mortality in patients undergoing pancreaticoduodenectomy for pancreatic adenocarcinoma. Updates Surg. 2023; 75:523–30. 10.1007/s13304-022-01404-4. 36309940 PMC10042927

[83] Moher D, Liberati A, Tetzlaff J, Altman DG, and PRISMA Group. Preferred reporting items for systematic reviews and meta-analyses: the PRISMA Statement. Open Med. 2009; 3:e123–30. 21603045 PMC3090117

[84] Sampson M, McGowan J, Cogo E, Grimshaw J, Moher D, Lefebvre C. An evidence-based practice guideline for the peer review of electronic search strategies. J Clin Epidemiol. 2009; 62:944–52. 10.1016/j.jclinepi.2008.10.012. 19230612

[85] Wells G, Shea B, O’Connell D, Peterson J, Welch V, Losos M. The Newcastle-Ottawa Scale (NOS) for assessing the quality of nonrandomized studies in meta-analyses. https://www.ohri.ca/. 2021. http://www.ohri.ca/programs/clinical_epidemiology/oxford.asp.

