# Supplementary material for: Incidence and risk factors for new-onset diabetes mellitus (NODM) in post-pancreatectomy patients diagnosed with pancreatic adenocarcinoma (PAC): A systematic review
Source: Oncoscience. 2025 Dec 8;12:203–18. doi: 10.18632/oncoscience.637 (PMC12687554; doi:10.18632/oncoscience.637)
Supplement: Supplementary file 1 [file oncoscience-12-637-s001.pdf]

Incidence and risk factors for new-onset diabetes mellitus (NODM) in post-pancreatectomy patients diagnosed with pancreatic adenocarcinoma (PAC): A systematic review

SUPPLEMENTARY MATERIALS

Supplementary File 1

Quality assessment in the methods

Methods

We performed the critical appraisal using the Newcastle-Ottawa Quality Assessment Scale for cohort studies [13].

Quality assessment for Cohort studies:

Sl. No.	Study ID	Representative of the exposed cohort	Selection of the non-exposed or less-exposed cohort	Ascertainment of exposure	Demonstration that the outcome of interest was not present at the start of study	Comparability	Assessment of outcome	Time of follow-up	Adequacy of follow up	Total score (stars)
1	Kanwat et al. [18]	1	0	1	1	0	1	1	1	6
2	Niwano et al. [19]	1	0	1	1	0	1	1	1	6
3	Maxwell et al. [20]	1	0	1	1	1	1	1	1	7
4	Singh et al. [21]	1	0	1	1	0	1	1	1	6
5	Yun et al. [22]	1	0	1	1	0	1	1	1	6
6	Pannala et al. [23]	1	1	1	1	1	1	1	1	8
7	Wu et al. [24]	1	0	1	1	0	1	1	1	6
8	Min Oh et al. [25]	1	1	1	1	1	1	1	1	8
9	Ferrera et al. [26]	1	1	1	1	1	1	1	1	8
10	Bock et al. [27]	1	0	1	1	0	1	1	1	6
11	You et al. [28]	1	0	1	1	0	1	1	1	6
12	Imamura et al. [29]	1	0	1	1	0	1	1	1	6
13	Tariq et al. [30]	1	1	1	1	0	1	1	1	7
14	Kang et al. [31]	1	0	1	1	0	1	1	1	6
15	Shirakawa et al. [32]	1	0	1	1	0	1	1	1	6
16	Shen et al. [33]	1	0	1	1	0	1	1	1	6
17	Izumo et al. [34]	1	0	1	1	0	1	1	1	6
18	Wu et al. [35]	1	1	1	1	1	1	1	1	8
19	Kwon et al. [36]	1	1	1	1	1	1	1	1	8
20	Malleo et al. [37]	1	1	1	1	0	1	1	1	7
21	Stuthfield et al. [38]	1	0	1	1	0	1	1	0	5
22	Chari et al. [39]	1	0	1	1	0	1	1	0	5
23	Adam et al. [40]	1	0	1	1	1	1	1	1	7
24	Lee et al. [41]	1	1	1	1	1	1	1	1	8
25	Ishida et al. [42]	1	1	1	1	1	1	1	1	8

26	Shaw et al. [43]	1	1	1	1	1	1	1	1	8
27	Niwano et al. [44]	1	1	1	1	1	1	1	1	8
28	Maxwell et al. [45]	1	1	1	1	1	1	1	1	8
29	Maignan et al. [46]	1	1	1	1	0	1	0	1	6
30	Wang et al. [47]	1	1	1	1	1	1	1	1	8
31	Yoo et al. [48]	1	1	1	1	1	1	1	1	8
32	Schranz et al. [49]	1	1	1	1	1	1	1	1	8
33	Thomas et al. [50]	1	1	1	1	1	1	1	1	8
34	Hamad et al. [51]	1	1	1	1	1	1	1	1	8
35	Yamada et al. [52]	1	1	1	1	1	1	1	1	8
36	Yamamoto et al. [53]	1	1	1	1	1	1	1	1	8
37	Mayeux et al. [54]	1	1	1	1	1	1	1	1	8
38	Lee et al. [55]	1	1	1	1	1	1	1	1	8
39	Kusakabe [56]	1	1	1	1	1	1	1	1	8
40	Karlin et al. [57]	1	1	1	1	0	1	1	1	7
41	Nguyen et al. [58]	1	1	1	1	1	1	1	1	8
42	Elliott et al. [59]	1	1	1	1	1	1	1	1	8
43	Burkhart et al. [60]	1	1	1	1	1	1	1	1	8
44	Hirata et al. [61]	1	1	1	1	1	1	1	1	8
45	White et al. [62]	1	1	1	1	0	1	1	1	7

Supplementary File 2

A. Incidence rates

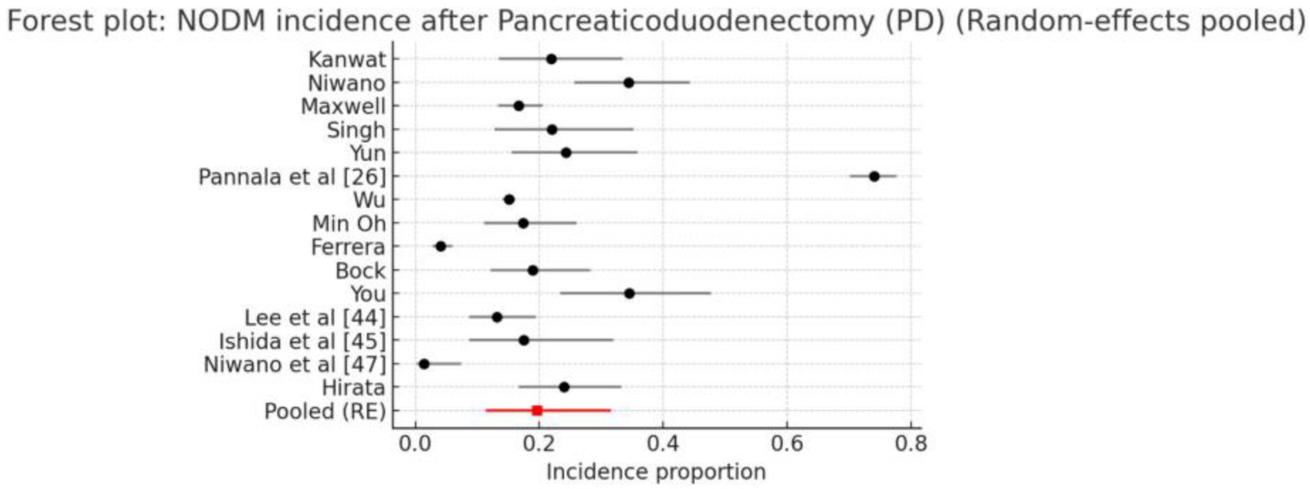

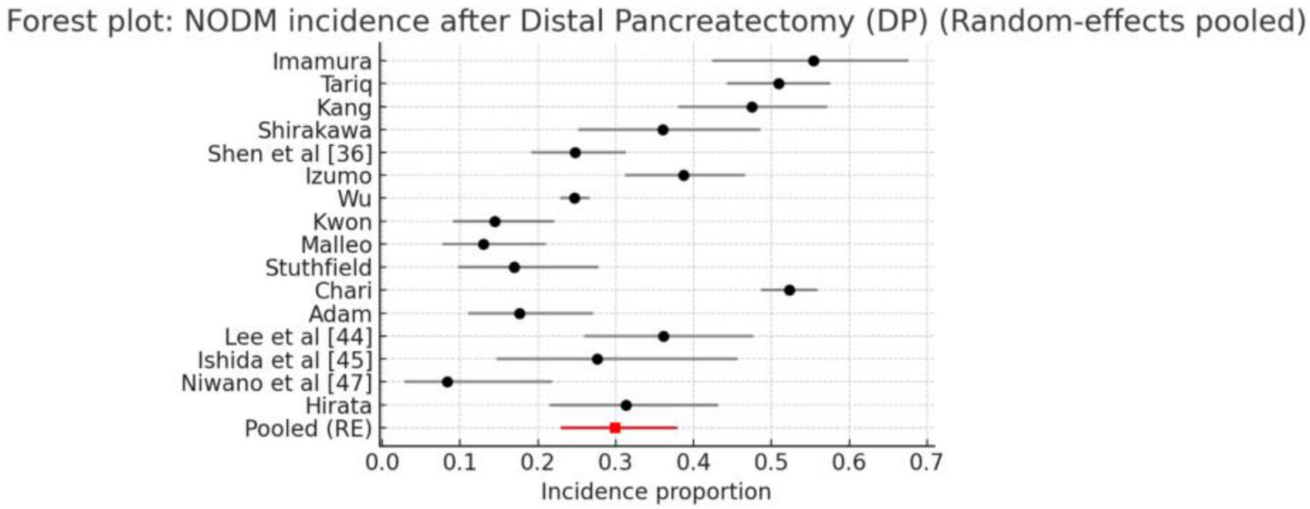

For Pancreatico-Duodenectomy (PD) group, the pooled incidence is 23.2% with 95% CI: 11.4% to 31.6%, with large heterogeneity.

For Distal pancreatectomy (DP) group, the pooled incidence is 26.3% with 95% CI: 22.9% to 37.9% with moderate heterogeneity.

B. Risk factor analysis

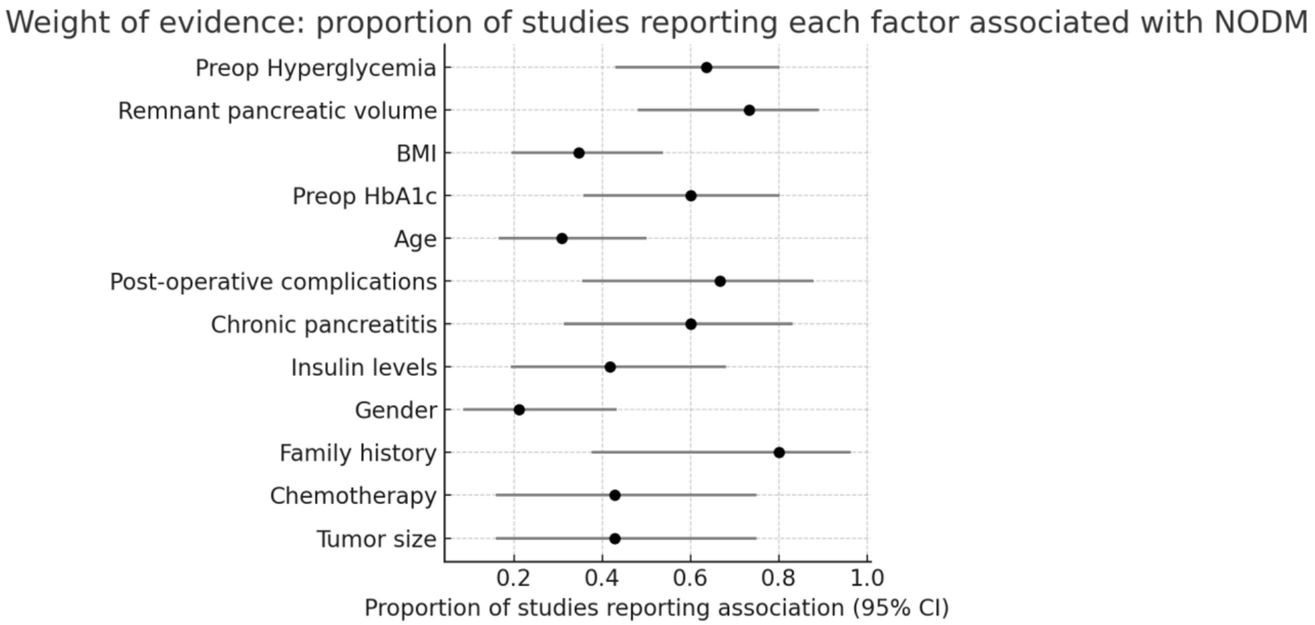

Factors with the strongest weight of evidence

1. Preoperative hyperglycemia — analysed in 22 studies; reported associated in 14 studies.
   Proportion = 64% (95% CI ≈ 46–78%).
2. Remnant pancreatic volume — analysed in 15; reported associated in 11.
   Proportion = 73% (95% CI ≈ 46–89%).
3. BMI — analysed in 26; reported associated in 9.
   Proportion = 35% (95% CI ≈ 21–52%).
4. Preop HbA1c — analysed in 15; reported assoc in 9.
   Proportion = 60% (95% CI ≈ 36–79%).
5. Age — analysed in 26; reported assoc in 8.
   Proportion = 31% (95% CI ≈ 18–47%).
6. Post-operative complications (e.g., POPF/infections) — analysed in 9; reported assoc in 6.
   Proportion = 67% (95% CI ≈ 37–88%).
7. Chronic pancreatitis — analysed in 10; reported assoc in 6.
   Proportion = 60% (95% CI ≈ 31–83%).

Supplementary File 3

Search strategy

The detailed search strategy for the included studies, formatted according to the Peer Review of Electronic Search Strategies (PRESS) guidelines as follows

PubMed Search Strategy

((“diabetes mellitus”[MeSH Terms] OR (“diabetes”[All Fields] AND “mellitus”[All Fields]) OR “diabetes mellitus”[All Fields]) AND (“pancreatic neoplasms”[MeSH Terms] OR (“pancreatic”[All Fields] AND “neoplasms”[All Fields]) OR “pancreatic neoplasms”[All Fields] OR (“pancreatic”[All Fields] AND “cancer”[All Fields]) OR “pancreatic cancer”[All Fields]) AND (“pancreatectomy”[MeSH Terms] OR “pancreatectomy”[All Fields] OR “pancreatectomies”[All Fields])) AND (systematicreview[Filter])
Translations
diabetes mellitus: “diabetes mellitus”[MeSH Terms] OR (“diabetes”[All Fields] AND “mellitus”[All Fields]) OR “diabetes mellitus”[All Fields]
pancreatic cancer: “pancreatic neoplasms”[MeSH Terms] OR (“pancreatic”[All Fields] AND “neoplasms”[All Fields]) OR “pancreatic neoplasms”[All Fields] OR (“pancreatic”[All Fields] AND “cancer”[All Fields]) OR “pancreatic cancer”[All Fields]
pancreatectomy: “pancreatectomy”[MeSH Terms] OR “pancreatectomy”[All Fields] OR “pancreatectomies”[All Fields]
